# ActiviDaily: Turning Apathy Into Action in Neurodegenerative Disease

**DOI:** 10.1093/geroni/igaa057.2909

**Published:** 2020-12-16

**Authors:** Lauren Massimo, Sean Lydon, Alexander Miller, Katya Rascovsky, Dawn Mechanic-Hamilton

**Affiliations:** University of Pennsylvania, Philadelphia, Pennsylvania, United States

## Abstract

Impairment of goal-directed behavior (GDB), often labeled apathy, is a common behavioral symptom in dementia. ActiviDaily is a novel mobile app that engages both patients and caregivers to increase GDB to improve everyday function. ActiviDaily targets key components of GDB (motivation, planning and initiation) and individualizes patient goals. Pilot testing in twelve patient/caregiver dyads occurred over 4 weeks of app use. Measures of behavior, everyday functioning, and psychological distress were assessed in a pre-post design. Goal Attainment Scaling (GAS) was used to establish individualized goals and measure progress on a standard scale. GAS showed that 79% of participants’ goals were met at or above expectations. Caregiver depression and stress were significantly reduced. There was also a reduction in ratings of patient apathy. ActiviDaily is an innovative intervention that individualizes treatment of apathy and has the potential to increase independence in day-to-day life and decrease caregiver burden.

